# Supplementary material for: Pyruvate Dehydrogenase Kinase 1 inhibition mediated oxidative phosphorylation enhancement in cartilage promotes osteoarthritis progression
Source: BMC Musculoskelet Disord. 2023 Jul 20;24:597. doi: 10.1186/s12891-023-06585-6 (PMC10357736; doi:10.1186/s12891-023-06585-6)
Supplement: Supplementary file 1 — Additional file 1. [file 12891_2023_6585_MOESM1_ESM.zip › KEGG.Gsea.1653622432464/KEGG_TASTE_TRANSDUCTION.html]

Details for gene set KEGG\_TASTE\_TRANSDUCTION[GSEA]

|  || Dataset | 55457\_collapsed\_to\_symbols.cls.cls #OA\_versus\_normal.cls.cls #OA\_versus\_normal\_repos |
| Phenotype | cls.cls#OA\_versus\_normal\_repos |
| Upregulated in class | normal |
| GeneSet | KEGG\_TASTE\_TRANSDUCTION |
| Enrichment Score (ES) | -0.20550187 |
| Normalized Enrichment Score (NES) | -0.7143859 |
| Nominal p-value | 0.8682842 |
| FDR q-value | 1.0 |
| FWER p-Value | 1.0 |
Table: GSEA Results Summary

  

Fig 1: Enrichment plot: KEGG\_TASTE\_TRANSDUCTION      
 Profile of the Running ES Score & Positions of GeneSet Members on the Rank Ordered List

  

| SYMBOL | TITLE | RANK IN GENE LIST | RANK METRIC SCORE | RUNNING ES | CORE ENRICHMENT || 1 | PRKACB | protein kinase cAMP-activated catalytic subunit beta [Source:HGNC Symbol;Acc:HGNC:9381] | 771 | 0.576 | 0.0209 | No |
| 2 | TAS2R13 | taste 2 receptor member 13 [Source:HGNC Symbol;Acc:HGNC:14919] | 813 | 0.565 | 0.0971 | No |
| 3 | PDE1A | phosphodiesterase 1A [Source:HGNC Symbol;Acc:HGNC:8774] | 1074 | 0.500 | 0.1471 | No |
| 4 | GNAT3 | G protein subunit alpha transducin 3 [Source:HGNC Symbol;Acc:HGNC:22800] | 2908 | 0.260 | 0.0411 | No |
| 5 | GNB3 | G protein subunit beta 3 [Source:HGNC Symbol;Acc:HGNC:4400] | 3800 | 0.177 | -0.0033 | No |
| 6 | GNB1 | G protein subunit beta 1 [Source:HGNC Symbol;Acc:HGNC:4396] | 3969 | 0.158 | 0.0059 | No |
| 7 | TAS2R9 | taste 2 receptor member 9 [Source:HGNC Symbol;Acc:HGNC:14917] | 4349 | 0.124 | -0.0061 | No |
| 8 | ASIC2 | acid sensing ion channel subunit 2 [Source:HGNC Symbol;Acc:HGNC:99] | 5652 | 0.019 | -0.1046 | No |
| 9 | TAS2R16 | taste 2 receptor member 16 [Source:HGNC Symbol;Acc:HGNC:14921] | 5763 | 0.011 | -0.1116 | No |
| 10 | GNG3 | G protein subunit gamma 3 [Source:HGNC Symbol;Acc:HGNC:4405] | 6079 | -0.011 | -0.1346 | No |
| 11 | TAS2R8 | taste 2 receptor member 8 [Source:HGNC Symbol;Acc:HGNC:14915] | 6268 | -0.025 | -0.1457 | No |
| 12 | TAS2R14 | taste 2 receptor member 14 [Source:HGNC Symbol;Acc:HGNC:14920] | 6516 | -0.043 | -0.1588 | No |
| 13 | TAS2R7 | taste 2 receptor member 7 [Source:HGNC Symbol;Acc:HGNC:14913] | 6592 | -0.049 | -0.1577 | No |
| 14 | ADCY8 | adenylate cyclase 8 [Source:HGNC Symbol;Acc:HGNC:239] | 6646 | -0.053 | -0.1544 | No |
| 15 | PLCB2 | phospholipase C beta 2 [Source:HGNC Symbol;Acc:HGNC:9055] | 7052 | -0.084 | -0.1741 | No |
| 16 | KCNB1 | potassium voltage-gated channel subfamily B member 1 [Source:HGNC Symbol;Acc:HGNC:6231] | 7193 | -0.096 | -0.1716 | No |
| 17 | SCNN1A | sodium channel epithelial 1 subunit alpha [Source:HGNC Symbol;Acc:HGNC:10599] | 7216 | -0.097 | -0.1597 | No |
| 18 | TAS2R1 | taste 2 receptor member 1 [Source:HGNC Symbol;Acc:HGNC:14909] | 7354 | -0.106 | -0.1554 | No |
| 19 | GRM4 | glutamate metabotropic receptor 4 [Source:HGNC Symbol;Acc:HGNC:4596] | 7658 | -0.131 | -0.1605 | No |
| 20 | CACNA1A | calcium voltage-gated channel subunit alpha1 A [Source:HGNC Symbol;Acc:HGNC:1388] | 7774 | -0.140 | -0.1499 | No |
| 21 | GNG13 | G protein subunit gamma 13 [Source:HGNC Symbol;Acc:HGNC:14131] | 8491 | -0.193 | -0.1784 | Yes |
| 22 | TAS2R4 | taste 2 receptor member 4 [Source:HGNC Symbol;Acc:HGNC:14911] | 8811 | -0.218 | -0.1726 | Yes |
| 23 | TAS2R10 | taste 2 receptor member 10 [Source:HGNC Symbol;Acc:HGNC:14918] | 8924 | -0.226 | -0.1496 | Yes |
| 24 | GNAS | GNAS complex locus [Source:HGNC Symbol;Acc:HGNC:4392] | 8929 | -0.226 | -0.1182 | Yes |
| 25 | SCNN1B | sodium channel epithelial 1 subunit beta [Source:HGNC Symbol;Acc:HGNC:10600] | 8945 | -0.227 | -0.0875 | Yes |
| 26 | CACNA1B | calcium voltage-gated channel subunit alpha1 B [Source:HGNC Symbol;Acc:HGNC:1389] | 9092 | -0.239 | -0.0652 | Yes |
| 27 | TAS2R3 | taste 2 receptor member 3 [Source:HGNC Symbol;Acc:HGNC:14910] | 10194 | -0.340 | -0.1031 | Yes |
| 28 | ITPR3 | "inositol 1,4,5-trisphosphate receptor type 3 [Source:HGNC Symbol;Acc:HGNC:6182]" | 10390 | -0.360 | -0.0678 | Yes |
| 29 | PRKACG | protein kinase cAMP-activated catalytic subunit gamma [Source:HGNC Symbol;Acc:HGNC:9382] | 10466 | -0.370 | -0.0217 | Yes |
| 30 | PRKACA | protein kinase cAMP-activated catalytic subunit alpha [Source:HGNC Symbol;Acc:HGNC:9380] | 10611 | -0.384 | 0.0211 | Yes |
| 31 | PRKX | protein kinase X-linked [Source:HGNC Symbol;Acc:HGNC:9441] | 11634 | -0.522 | 0.0149 | Yes |
| 32 | ADCY6 | adenylate cyclase 6 [Source:HGNC Symbol;Acc:HGNC:237] | 12005 | -0.594 | 0.0695 | Yes |
Table: GSEA details [plain text format]

  

Fig 2: KEGG\_TASTE\_TRANSDUCTION      
 Blue-Pink O' Gram in the Space of the Analyzed GeneSet

  

Fig 3: KEGG\_TASTE\_TRANSDUCTION: Random ES distribution      
 Gene set null distribution of ES for **KEGG\_TASTE\_TRANSDUCTION**
